# Context Mediates Antimicrobial Efficacy of Kinocidin Congener Peptide RP-1

**DOI:** 10.1371/journal.pone.0026727

**Published:** 2011-11-04

**Authors:** Nannette Y. Yount, Samuel E. Cohen, Deborah Kupferwasser, Alan J. Waring, Piotr Ruchala, Shantanu Sharma, Karlman Wasserman, Chun-Ling Jung, Michael R. Yeaman

**Affiliations:** 1 Division of Infectious Diseases, Los Angeles County-Harbor University of California Los Angeles Medical Center, Torrance, California, United States of America; 2 Los Angeles Biomedical Research Institute at Harbor-University of California Los Angeles Medical Center, Torrance, California, United States of America; 3 School of Medicine, University of California Irvine, Irvine, California, United States of America; 4 Division of Pulmonary / Critical Care Medicine, Los Angeles County-Harbor University of California Los Angeles Medical Center, Torrance, California, United States of America; 5 Department of Medicine, David Geffen School of Medicine at University of California Los Angeles, Los Angeles, California, United States of America; 6 Department of Physiology and Biophysics, School of Medicine, University of California Irvine, Irvine, California, United States of America; 7 Materials and Process Simulation Center, California Institute of Technology, Pasadena, California, United States of America; 8 Division of Molecular Medicine, Los Angeles County-Harbor University of California Los Angeles Medical Center, Torrance, California, United States of America; University of Rome, Italy

## Abstract

Structure-mechanism relationships are key determinants of host defense peptide efficacy. These relationships are influenced by anatomic, physiologic and microbiologic contexts. Structure-mechanism correlates were assessed for the synthetic peptide RP-1, modeled on microbicidal domains of platelet kinocidins. Antimicrobial efficacies and mechanisms of action against susceptible (^S^) or resistant (^R^) *Salmonella typhimurium* (ST), *Staphylococcus aureus* (SA), and *Candida albicans* (CA) strain pairs were studied at pH 7.5 and 5.5. Although RP-1 was active against all study organisms, it exhibited greater efficacy against bacteria at pH 7.5, but greater efficacy against CA at pH 5.5. RP-1 de-energized SA and CA, but caused hyperpolarization of ST in both pH conditions. However, RP-1 permeabilized ST^S^ and CA strains at both pH, whereas permeabilization was modest for ST^R^ or SA strain at either pH. Biochemical analysis, molecular modeling, and FTIR spectroscopy data revealed that RP-1 has indistinguishable net charge and backbone trajectories at pH 5.5 and 7.5. Yet, concordant with organism-specific efficacy, surface plasmon resonance, and FTIR, molecular dynamics revealed modest helical order increases but greater RP-1 avidity and penetration of bacterial than eukaryotic lipid systems, particularly at pH 7.5. The present findings suggest that pH– and target–cell lipid contexts influence selective antimicrobial efficacy and mechanisms of RP-1 action. These findings offer new insights into selective antimicrobial efficacy and context–specificity of antimicrobial peptides in host defense, and support design strategies for potent anti-infective peptides with minimal concomitant cytotoxicity.

## Introduction

Host defense effector molecules such as antimicrobial peptides are key contributors to first line defense against invading microorganisms. Presently, over 3,000 naturally-occurring cationic antimicrobial peptides have been isolated from organisms spanning the evolutionary continuum [1], [2]. Structurally, such peptides can be divided into two major groups: 1) linear / extended peptides predominantly of α-helical conformation; and 2) disulfide-containing peptides that largely exhibit β-sheet structures [1], [2]. Mechanisms of antimicrobial efficacy of host defense peptides can be generalized to involve three complementary modes: cytoplasmic membrane permeabilization, cytoplasmic membrane (bacterial) or mitochondrial (eukaryotic pathogen) de-energization, and inhibition of macromolecular synthesis (e.g. cell wall, nucleic acid) or essential pathways [2]. Such mechanisms are believed to involve initial peptide targeting based on electrostatic and hydrophobic interactions, followed by structural or functional organization of the peptide upon or within target cell or organelle membranes [3].

Host defense peptides interact with pathogens in distinct anatomic and physiologic settings. It follows that specific peptides have evolved for optimal function in respective host contexts against cognate pathogens. For example, neutrophil phagolysosomes or other inflammatory contexts exhibit acidic pH of 4.5 to 6.5 [4]. In contrast, the bloodstream and mucosal surfaces maintain neutral pH of 7.2 to 7.5 [5]. We hypothesize that host anatomic, physiologic, and microbiologic contexts shape peptide selective efficacy or immunomodulatory functions *in situ* [1], [6], [7], [8]. These conditional factors likely influence peptide selectivity for specific cell targets, such as anionic versus zwitterionic phospholipids, and prokaryotic versus eukaryotic differences in transmembrane potential (ΔΨ) [2].

Beyond fundamental characterization of a peptide, investigating its interactions with target pathogens provides new opportunities to understand specific mechanisms of action and selective toxicity. In the current investigation, this approach was integrated to assess the impact of pH and lipid context on pathogen-specific and structure-mechanism relationships of the synthetic anti-infective candidate peptide RP-1 against prototypical Gram-positive, Gram-negative, and fungal pathogens. The current studies were designed to address the hypothesis that pH and/or target cell-specific contexts, alone or in combination, impact antimicrobial peptide selectivity and efficacy.

## Results

### Influence of pH on RP-1 Antimicrobial Specificity

RP-1 exerted potent efficacy against ST, SA and CA under one or more experimental conditions (**Table 1**; also see **Table S1**). However, several functional correlates were observed reflecting pH and organism specificity. For example, RP-1 efficacy was greatest overall against bacteria at pH 7.5. Interestingly, RP-1 had equivalent efficacy against ST and SA strains at pH 7.5, with no significant differences between respective S and R strains in this condition. In contrast, pH 5.5 conditions disclosed significant differences in RP-1 efficacy against the S and R phenotypes of ST and SA strains. Moreover, RP-1 exerted greatest anti-candidal efficacy at pH 5.5. There were no detectable differences in RP-1 susceptibility of the CA^S^ and CA^R^ strains at a given pH value. Therefore, RP-1 target organism selectivity and efficacy were distinctive as a function of pH.

**Table 1:** Antimicrobial activity of RP-1 versus representative ST, SA and CA sensitive and resistant pathogen strain pairs.

	*S. typhimurium*		*S. aureus*		*C. albicans*	
pH	ST^S^	ST^R^	SA^S^	SA^R^	CA^S^	CA^R^
**5.5**	14.0±0.7†	8.8±0.4	7.0±0.9†	3.8±0.4	9.1±0.3	8.9±0.9
**7.5**	15.5±0.4*	14.5±0.7*	13.3±0.8*	12.8±1.8*	6.1±3.2	6.6±2.9

Antimicrobial efficacy is measured as the zone of inhibition (millimeters) around a central well. Significant differences (***P*** values≤0.05) within strain at pH 5.5 versus 7.5 (†); or between sensitive and resistant strains at constant pH (*).

### Influence of pH and Target Cell Specificity on RP-1 Mechanisms of Action

**Figure 1:**
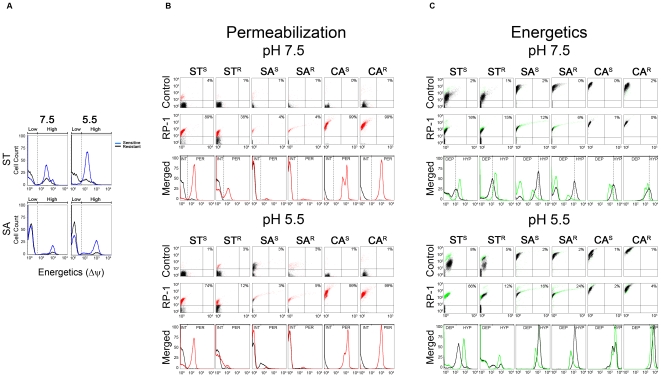
Flow cytometric analysis of: (A) basal membrane electronegativity; and (B) membrane permeabilization or (C) energetics for untreated and RP-1 treated cell populations at pH 7.5 and 5.5. (A) Basal membrane electronegativity (untreated stained cells) for sensitive versus resistant ST and SA at pH 7.5 and 5.5. Comparison of membrane permeabilization (B) or energetics (C) between untreated (black) and RP-1 treated microorganisms. RP-1 and Control panels (B,C) depict fluorescence (Y-axis) vs. FSC (forward scatter; size / granularity; X-axis) of individual cells. Indicated values represent the percentage of cells that are beyond the control (black) area. Merged panel shows histogram analysis of: (B) permeabilization - control (black) vs. RP-1 treated (red); or (C) energetics - control (black) vs. RP-1 treated (green). INT = intact cells; PER = permeabilized cells; DEP = depolarized cells; HYP = hyperpolarized cells.

As a complement to efficacy data, the impact of pH and target specificity was studied for two comparative mechanisms of RP-1: membrane permeabilization (MP) and ΔΨ (**Figure 1**; also see **Figure S1**).

### RP-1 vs. S. typhimurium

The ST study strains are known to differ in ΔΨ, conferring distinct susceptibility profiles to certain cationic peptides. Consistent with this relationship, basal membrane ΔΨ was reduced for ST^R^ versus ST^S^ (**Figure 1A**). Flow cytometry corroborated significant RP-1 efficacy against ST strains at pH 7.5. RP-1 exerted virtually complete MP of ST^S^ at pH 7.5 or 5.5, but modest MP of ST^R^ at pH 7.5, and minimal MP at pH 5.5 (**Figure 1**B). Interestingly, RP-1 exposure led to increased ΔΨ (hyperpolarization) of both ST strains at pH 7.5, but only the ST^R^ strain at pH 5.5 (**Figure 1C**).

### RP-1 vs. S. aureus

Present studies revealed that the basal ΔΨ of the SA^R^ strain was lower than its SA^S^ counterpart, consistent with prior findings (**Figure 1A** **;** [**9**]). RP-1 exposure led to alterations in ΔΨ and permeabilization in SA that differed from those for ST. For example, RP-1 induced minimal MP of either SA strain at pH 5.5 or 7.5 over 1 h exposure (**Figure 1B**). Nonetheless, RP-1-induced depolarization of SA strains was equivalent under either pH condition over the assay period (**Figure 1C**).

### RP-1 vs. C. albicans

RP-1-induced permeabilization of CA strains was significant and equivalent at pH 5.5. as well as 7.5 (**Figure 1B**). In contrast, depolarization was minimal for either CA strain at either pH (**Figure 1C**) over the time period evaluated.

### Influence of pH on RP-1 Physicochemistry

**Figure 2:**
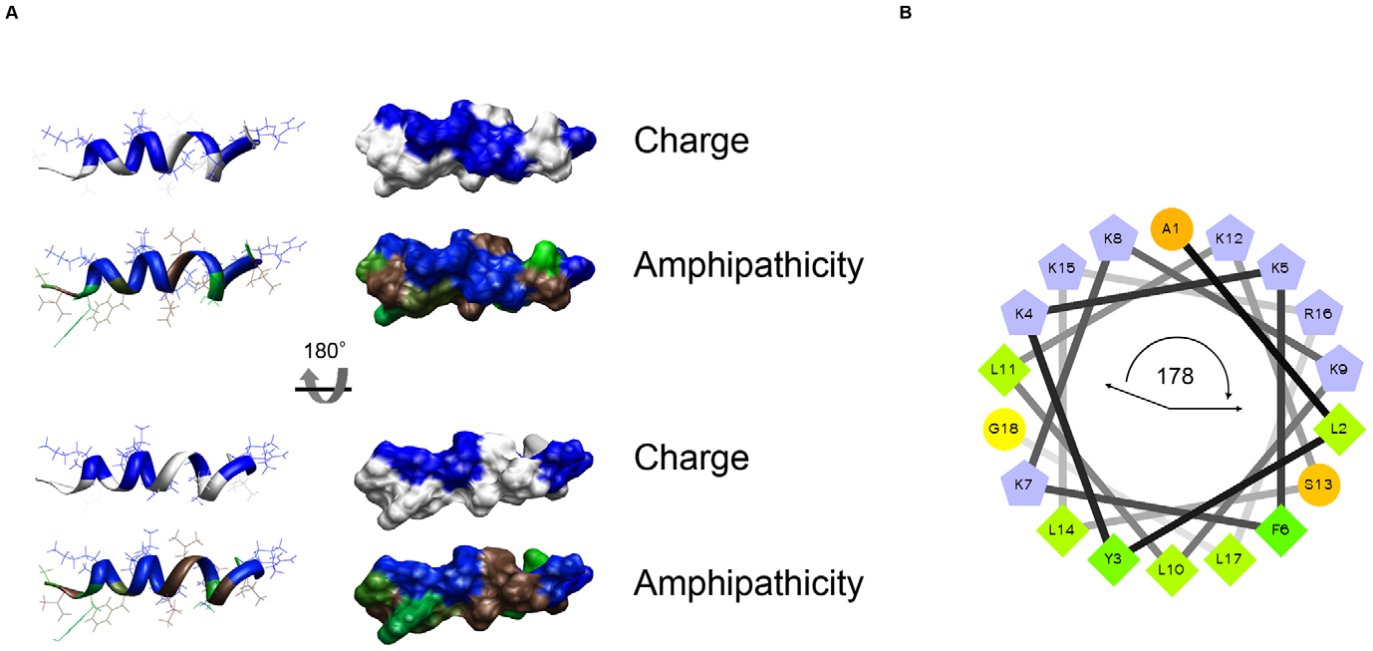
Structure and biophysical properties of RP-1. (A) Ribbon and space-filling representations of RP-1. Charge: blue, basic (Arg, Lys) cationic residues. Amphipathicity: Kyte-Doolittle hydropathy plot; brown, most hydrophobic; green intermediate; blue most hydrophilic. Molecular imaging by Chimera [52]. (B) Helical wheel representation with polar angle subtended by hydrophilic residues as indicated.

Given the differences observed in RP-1 target organism selectivity and efficacy as a function of pH, potential effects of pH on RP-1 physicochemistry were assessed. RP-1 has a net charge of +7.7 at pH 7.5, with a partial charge increase calculated to +8.0 at pH 5.5 (**Table 2**). Based on its NMR-determined 3-D structure, RP-1 conforms to a highly-ordered amphipathic α-helix [10] **Figure 2A**). Such a conformation segregates electrostatic charge and hydrophobicity to relative contralateral facets of the peptide, yielding a polar angle of 178.6° (**Figure 2B**). At pH 7.5, the calculated mean hydrophobic moment (M_H_) for RP-1 is 0.66, with a net hydrophobicity of −1.45. No distinct impact of pH 5.5 on these parameters of RP-1 was detected in the current studies.

**Table 2:** Sequence and biophysical properties of RP-1.

				Calculated Charge at pH					
Peptide	Sequence	MW	pI	5.0	5.5	6.0	6.5	7.0	7.5
RP-1	A^1^LYKK^5^FKKKL^10^LKSLK^15^RLG^18^	2162.7	10.8	8.0	8.0	8.0	8.0	7.9	7.7

### Influence of pH and Target Lipid Context on RP-1 Structure-Activity Relationships

To integrate pH and target lipid context influences on RP-1 structure-activity relationships, multiple and complementary approaches were used to compare peptide secondary structure and orientation in mimetic liposome systems reflecting bacterial and eukaryotic membrane systems [11]. Several key findings emerged from these investigations.

### Influence of pH on RP-1 Affinity for Bacterial versus Mammalian Lipid Systems

**Figure 3:**
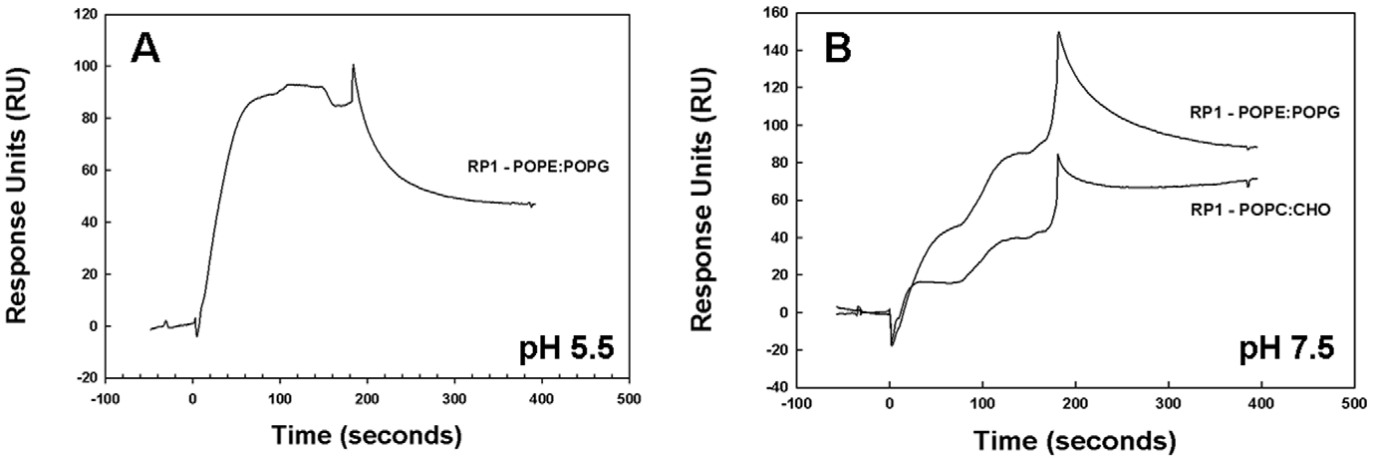
SPR analysis of RP-1 membrane interactions using bacterial and eukaryotic membrane mimetic systems. (A) Response units for association of RP-1 with POPE∶POPG (3∶1) or POPC∶CHO (1.2∶1) immobilized lipid multilayers at pH 5.5. Note: SPR was not detectable for the POPC∶CHO system at pH 5.5. (B) SPR data for RP-1 interaction with the membrane systems described above at pH 7.5.

Kinetic interactions of RP-1 with membrane liposomes were measured by two complementary methods. Importantly, surface plasmon resonance (SPR) analysis demonstrated strongest association between RP-1 and bacterial lipids at pH 7.5 (K_D_ = 1.15×10^−7^ M). By comparison, the affinity of RP-1 for eukaryotic mimetic lipids was markedly reduced at this pH (K_D_ = 5.29×10^−7^ M). Moreover, RP-1 also significantly associated with prokaryotic lipids at pH 5.5 (K_D_ = 9.18×10^−8^ M), while there was no detectable interaction of RP-1 and eukaryotic lipids at this pH (**Table 3**). In addition, the temporal association of RP-1 with lipid systems varied with pH. Interestingly, at pH 7.5 there was a multi-phasic interaction between RP-1 and both target lipid ensembles (**Figure 3**). In contrast, at pH 5.5 the association of RP-1 with bacterial lipids was relatively mono-phasic, while it was undetectable for eukaryotic lipids.

**Table 3:** RP-1 interaction with lipid systems by quantitative FTIR spectroscopy.

Lipid System	pH	% Conformation				Tilt Angle Θ	K_A_ (M^−1^)	K_D_ (M)
		α-helix	loop-turn	β-sheet	disordered			
**POPE∶POPG**	7.5	51.5	31.6	6.5	10.4	51°	5.3×10^4^	1.2×10^−7^
**POPE∶POPG**	5.5	50.2	32.9	8.7	8.2	46°	1.3×10^4^	9.2×10^−8^
**POPC∶CHO**	7.5	42.9	33.8	13.2	10.1	34°	0.3×10^4^	5.3×10^−7^
**POPC∶CHO**	5.5	40.2	34.2	14.9	10.7	31°	0.1×10^4^	ND

The secondary structure and orientation of RP-1 was determined using representative bacterial (POPE/POPG; mole ratio 3∶1) and eukaryotic (POPC/cholesterol; mole ratio 1.2∶1) mimetic liposomal systems. For conformation assessment lipid-peptide films were dispersed in deuterated buffer (10 mM PIPES pH 5.5 or 10 mM HEPES pH 7.5) and FTIR spectra of the samples were averaged for 256 scans at a gain of 4 and a resolution of 2 cm−1. The relative amounts of α-helix, β-turn, β-sheet, and random (disordered) structures were estimated by Fourier self-deconvolution and the tabulated results represent means from four independent and highly reproducible determinations for each environment SE 5%, or better. K_assoc_ was measured by introducing RP-1 to solutions containing large unilamellar vesicles (10 mM PIPES pH 5.5 or 10 mM HEPES pH 7.5). The orientation of the RP-1 peptide in the lipid bilayer of eukaryotic and bacterial membrane mimetic systems was determined using polarization (0° to 90°) to determine the insertion, or tilt angle of the peptide helical axis in the lipid multilayers. The binding of the peptide to lipid was expressed as an association constant ***K_assoc_ [1]***, where ***[P]*** is the molar concentration of RP-1 peptide in solution, ***[L]*** is the molar concentration of lipid and ***[PL]*** is the molar concentration of peptide bound to lipid [39].

### Binding Kinetics

Binding studies showed that RP-1 has significantly greater affinity for prokaryotic than eukaryotic lipids, particularly at pH 5.5 (**Figure 3**). In agreement with efficacy and SPR outcomes, K_A_ was greatest between RP-1 and bacterial large unilamellar vesicles (LUVs) at pH 7.5 (**Table 3**). Moreover, K_A_ was significantly reduced for RP-1 with the same membrane lipids at pH 5.5. Interestingly, the association between RP-1 and a mammalian lipid system was minimal at pH 5.5. Overall, these data are concordant with antimicrobial efficacy outcomes.

### Conformation

Conformation was measured using FTIR spectroscopy and relative amounts of α-helix (1662–1645 cm^−1^), β-sheet (1637–1613 and 1710–1682 cm^−1^), turn/bend (1682–1662 cm^−1^), and disordered or random (1650–1637 cm^−1^) structures were estimated by Fourier self-deconvolution. Component peak areas were calculated using curve fitting software. These analyses suggest that RP-1 may be slightly more helical in bacterial mimetic lipids, with 52% helicity at pH 7.5 and 50% helicity at pH 5.5 (**Table 3** **; Figure S2**). In contrast, in this mammalian lipid system, RP-1 exhibited less helical propensity, with 43% and 40% helicity at pH 7.5 and 5.5 respectively. In each case, relative β-sheet constituents reflected the modest differences corresponding to α-helical content in the distinct lipid environments.

### Angle of Insertion

The angle of RP-1 insertion in prokaryotic or eukaryotic membrane systems was estimated by FTIR absorbance at 0° and 90° polarization (**Table 3**). These experiments indicated that the greatest degree of helical tilt for RP-1 (51°) occurred in bacterial lipid multilayers at pH 7.5. Reducing the pH to 5.5 yielded a 5° reduction in the RP-1 helical tilt angle (46°). This difference in insertion angle corresponded with the peptide spanning the bilayer leaflet interface at pH 7.5, but not at pH 5.5 (see below). Notably, the RP-1 helical tilt angle was markedly less acute (34° or 31° at pH 7.5 or 5.5, respectively) upon peptide interaction with zwitterionic, cholesterol containing membranes (**Table 3**).

### Molecular Dynamics

**Figure 4:**
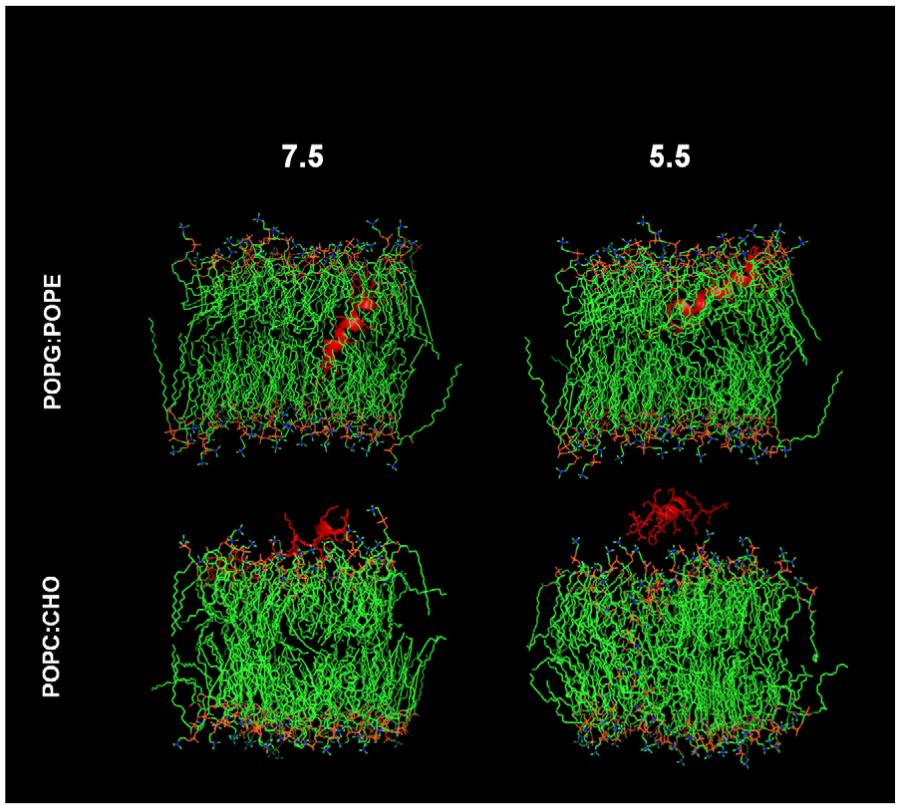
Molecular dynamic simulation of RP-1 interactions with representative bacterial versus eukaryotic lipid systems at pH 7.5 and 5.5. RP-1 was docked onto the polar head group domain of a pre-equilibriated POPE∶POPG (3∶1, mole∶mole) bilayer, or POPC∶CHO (1∶1, mole∶mole) with Hyperchem 7.5. Pre-run dynamics were carried out for 20 psec at 311 K, followed by 10 nsec of molecular dynamics at 311 K. Molecular model structures were rendered using PyMOL v0.99 (http://www.pymol.org).

As a complement to empirical studies of efficacy, conformation and binding, *in-silico* studies of RP-1-lipid interactions were performed by molecular dynamics using the initial NMR (2RLG; [10]) structure consistent with conditions of pH 5.5. Consistent with FTIR data, RP-1 penetrated the bacterial lipid environment at a sharp angle (∼48°) and was completely embedded into the bilayer under these conditions (**Figure 4**). Moreover, this extent of lipid insertion was such that RP-1 spanned the outer-to-inner membrane leaflet interface. Further corroborating the biophysical data, the degree of lipid penetration was reduced in the bacterial lipid system at pH 5.5, with RP-1 restricted to the outer membrane leaflet. In marked contrast to prokaryotic lipids, simulations for RP-1 in eukaryotic membrane environments at pH 7.5 predicted little if any membrane penetration, and no detectable interaction at pH 5.5 (**Figure 4**). Under either pH condition, RP-1 assumed a predominantly random coil conformation, and was restricted to the phospholipid-water interface with little detectable lipid interaction.

To explore the hypothesis that pH may influence RP-1 interactions with the study lipids, a detailed structure analysis was performed comparing the present molecular dynamics data and prior NMR data. Amino acid side chain conformations of RP-1 in POPE/POPG or POPC/CHO in conditions approximating pH 5.5 or 7.5 were compared with those of previous NMR studies of the peptide in membrane mimic micelles (pH 5.0; [10]) are shown in **Table 4**. Amino acid side chain orientations in SDS and anionic lipids were similar. However, side chain orientations in mammalian membrane lipid bilayers were distinct from those of zwitterionic DPC, suggesting the degree of peptide interaction with the POPC∶CHO ensembles was different from that with the micelles in the NMR studies. Thus, the present data are consistent with prior NMR findings suggesting that pH may influence RP-1 side chain interactions, particularly in the bacterial lipid system, potentially contributing to context-specific efficacy.

**Table 4:** Comparison of RP-1 side chain conformations in membrane mimic environments using SCit side chain analysis.

ResidueNumber	SDS (pH 5.0)		POPE/POPG RP-1 (pH 5.5)		POPE/POPG RP-1 (pH 7.5)		DPC (pH 5.0)		POPC/CHO RP-1 (pH 5.5)		POPC/CHO RP-1 (pH 7.5)	
	Chi1	Chi2	Chi1	Chi2	Chi1	Chi2	Chi1	Chi2	Chi1	Chi2	Chi1	Chi2
**1 ALA**	NV	NV	NV	NV	NV	NV	NV	NV	NV	NV	NV	NV
**2 LEU**	29.8	122.1	29.6	122.1	−131.5	175.9	34.2	113.7	−144.6	−179.6	−144.6	−179.6
**3 TYR**	45.9	23.5	45.9	23.5	−77.8	105.3	58.6	70.0	−165.6	85.7	−165.6	85.7
**4 LYS**	60.4	−106.7	60.9	−106.3	50.2	−169.5	62.0	−104.2	−104.3	−75.3	−104.3	−75.3
**5 LYS**	148.6	−159.3	148.2	−159.8	−59.8	108.2	75.4	−143.8	−60.4	171.2	−60.4	171.2
**6 PHE**	−55.1	−1.4	−55.1	−1.5	−71.9	−108.1	70.4	51.2	−56.9	−84.8	−56.9	−84.8
**7 LYS**	83.6	92.6	83.6	92.6	−67.2	−179.9	172.9	−124.8	−82.5	176.8	−82.5	176.8
**8 LYS**	116.5	−111.2	116.2	−111.4	−69.6	152.2	−103.2	−116.7	−140.0	81.4	−140.0	81.4
**9 LYS**	145.1	96.6	145.1	96.6	−83.9	−60.2	−65.0	129.0	−71.7	−79.8	−71.7	−79.8
**10 LEU**	−157.4	111.5	−157.4	111.5	−62.0	177.6	−51.0	−51.0	−97.0	81.6	−97.0	81.6
**11 LEU**	92.3	81.1	92.3	81.1	−177.5	50.2	−50.4	177.2	−78.0	168.6	−78.0	168.6
**12 LYS**	141.1	−105.5	141.1	−105.5	49.3	−179.8	113.8	−128.0	−59.0	−73.7	−59.0	−73.7
**13 SER**	−23.0	NV	−23.0	NV	67.8	NV	170.7	NV	−59.2	NV	−59.2	NV
**14 LEU**	152.8	134.3	152.8	134.3	−58.4	172.6	126.0	142.5	−90.0	−57.8	−90.0	−57.8
**15 LYS**	170.9	123.1	170.9	123.1	−91.4	−57.8	−179.9	−85.9	−154.9	−135.8	−154.9	−135.8
**16 ARG**	130.3	66.0	−130.3	−66.0	−71.8	179.0	−175.3	34.2	−83.9	−172.9	−83.9	−172.9
**17 LEU**	68.9	83.4	68.9	83.4	−77.0	174.0	100.9	94.5	−71.6	−162.5	−71.6	−162.5
**18 GLY**	NV	NV	NV	NV	NV	NV	NV	NV	NV	NV	NV	NV

SDS and DPC estimates are derived from previously defined NMR structures (PDB accession codes 2RLH or 2RLG; pH 5.0 [10]). In the present studies, RP-1 side chain conformations were in simulated POPE/POPG and POPC/CHO lipid environments approximating protonating (pH 5.5) or non-protonating (pH 7.5) conditions. NV, no consistent value.

## Discussion

Host defense peptides are expressed in highly diverse anatomic and physiologic niches. Such expression is hypothesized to concatenate structure and function of a given peptide in-context to optimize efficacy against cognate pathogens. This concept is has been termed immunorelativity of host defense peptides [6]. Moreover, to have potential utility as anti-infective therapeutics, native or synthetic peptides must selectively function against target pathogens in context of infection without concomitant host cytotoxicity. An important barrier to development of novel anti-infective peptides has been suboptimal efficacy, durability, or toxicity in relevant host context. Thus, it is highly significant to define context-specific and pathogen selective toxicity and mechanisms of action of host defense peptides or synthetic congeners thereof.

Our prior studies have demonstrated RP-1 to exert rapid and potent microbicidal activities, with significantly greater efficacy in blood and blood matrices than in artificial media [12]. In contrast to many classical antimicrobial peptides, RP-1 has striking antimicrobial efficacy when administered intravenously in rigorous models of infection *in vivo*. For example, systemic RP-1 is highly efficacious alone or in combination with vancomycin in a murine model of 7 d-established *S. aureus* catheter biofilm infection [13]. Hence, RP-1 sufficiently differentiates between pathogen and host cell targets to achieve systemic efficacy without concomitant toxicity.

In the present investigation, pH- and target organism-specific efficacy, mechanisms of action, and lipid interactions of RP-1 were compared in prototypic Gram-positive and Gram-negative bacterial, and fungal pathogens, or mimetic systems representing prokaryotic and eukaryotic lipid contexts. Based on highly concordant results from integrative and complementary approaches, the current data indicate that pH- and/or target-specific contexts mediate peptide efficacy and mechanisms of action against respective microbial versus host cell targets. These outcomes suggest that pH and cellular lipid environments may induce subtle changes in peptide structure-function relationships resulting in robust effects favoring peptide interactions with pathogen rather than host cells.

A goal of the present FTIR studies was to assess if there were any gross conformational changes in RP-1 structure as influenced by lipid and/or pH context that may reflect a global conformational propensity. If such changes occurred, they might readily explain differences in RP-1 binding to lipids, or distinct antimicrobial effects at different pH. FTIR suggested modest yet detectable changes in peptide conformations in distinct membrane-like and pH contexts. In addition, a more detailed comparison of prior NMR [10] and current FTIR data was performed using molecular dynamics (**Table 4**). Prior solution NMR was conducted on a residue specific basis to compare RP-1 backbone trajectories in micelles (SDS, bacterial mimic system; or DPC, mammalian mimic system). In the current study, FTIR was used to evaluate RP-1 conformation in multilayer films. Results observed herein are consistent with prior NMR studies, providing a basis for the hypothesis that side chain conformations of RP-1 differ in bacterial vs. mammalian lipid context. If so, subtle variations in side chain conformation may be influenced by target lipid composition and pH, contributing to antimicrobial specificity and efficacy of RP-1.

Overall, microbial target, lipid membrane environment, and pH each contributed to RP-1 structure efficacy relationships. For example, prokaryotic versus eukaryotic lipid components had a discernable impact on overall peptide conformation. These data should be considered in context, recognizing that NMR and other methods have limitations regarding structural resolution of helical termini. RP-1 was determined to be more helical in prokaryotic bacterial lipid systems (anionic), than in zwitterionic eukaryotic lipid environments [10]. Further, RP-1 interactions were significantly stronger for bacterial rather than eukaryotic lipid systems. By comparison, the overall influence of pH on RP-1 structure-activity relationships appeared to be more complex. In molecular dynamic simulations, a reduction in pH from 7.5 to 5.5 decreased the angle of penetration in bacterial lipids. These findings corroborate the findings that RP-1 was more efficacious against bacteria at pH 7.5. However, pH did not appear to have a significant impact on the physicochemistry of RP-1 overall. These results suggest two additional possibilities for future investigation: 1) pH may cause subtle changes in side chain orientation that translates to different RP-1 efficacy as a function of pH [10]; and 2) pH may affect target organisms in such a way as to alter susceptibility to RP-1. In either case, the present findings support the view that efficacy of RP-1 and like peptides is a collective result of intrinsic structural and functional optimization in context of organism-specific targets and the microenvironment in which the two interact.

The Gram-negative ST strains used in the current studies differ in susceptibility to defensin family antimicrobial peptides [14]. The genetic deficiency in ST^S^ results from inactivation of the gene encoding the transcriptional regulator PhoP. PhoP disruption voids multiple transcriptionally-induced peptide resistance mechanisms, including protease elaboration and differences in membrane ΔΨ [2]. Interestingly, the current data revealed RP-1-induced hyperpolarization of the ST strains, which was somewhat greater at pH 5.5. Although not a main focus of this study, peptide induced hyperpolarization has also been observed for the antifungal peptides PAF [15] and Rs-AFP2 [16]. In general, the current studies also suggested that RP-1 permeabilized and inactivated the sensitive ST^S^ strain to greater degree than its resistant counterpart. Such observations suggest mechanisms of α-helical peptides such as RP-1 may not be differentiated by the ST^S^ vs. ST^R^ phenotypes, but are influenced by pH.

In Gram-positive SA organisms, RP-1 also had significantly greater efficacy at pH 7.5 versus 5.5. Equivalent efficacy was seen against the SA^S^ and SA^R^ strains at pH 7.5, but significant differences found at pH 5.5. Interestingly, RP-1 induced significant depolarization of both SA strains at pH 5.5 and 7.5. In contrast, little or no membrane permeabilization of SA was caused by RP-1 against either strain in 1 hr. Xiong *et al.* previously demonstrated that RP-1 is able to permeabilize SA after 2 h of exposure [17]. Together, these findings agree with a temporal process of RP-1 interaction with SA, in which initial membrane interactions lead to rapid dissipation of ΔΨ, which precedes MP. This scenario is entirely consistent with RP-1 inhibition of electron transport [9], induction of a selectively permeable channel or pore, inhibition of intracellular targets [17], or other perturbations of the cell membrane without overt disruption of its lipid bilayer. Resolving specific mechanisms of RP-1 action and selective toxicity is a focus of ongoing studies. However, the current data point to the initial interaction between RP-1 and SA, as influenced by strain phenotype and context factors such as pH, as being integral to selective targeting and ensuing efficacy.

Comparative efficacy and mechanisms of action in the eukaryotic pathogen CA was of direct relevance to the hypothesis that pH and lipid context impact RP-1 selective toxicity. In contrast to bacteria, RP-1 exerted greater anti-fungal efficacy at pH 5.5 than 7.5. The CA strains used in this study differ in expression of the *SSD1* gene product [18]. While its precise role in peptide resistance is under investigation, *SSD1* hyper-expression is thought to confer resistance to certain host defense peptides through altered CA envelope structure and mitochondrial protection. We previously found depolarization can precede later increases in phosphatidylserine accessibility during apoptotic-like killing by host defense peptides in *C. albicans* [19]. Thus, the fungicidal consequences of RP-1 are governed by events subsequent to initial cell membrane interactions, including access to and inhibition of fungal mitochondria. Supporting this concept, Helmerhorst *et al* [20], Gyurko *et al* [21], and Jang *et al* [22] have shown certain host defense peptides to enter CA species and inhibit crucial intracellular targets, including mitochondria.

Biophysical experiments demonstrated the phospholipid context to have a significant impact on RP-1 conformation, angle of penetration, and kinetic interactions with respective target lipids. Concordant FTIR, UV spectroscopy, SPR, and molecular dynamics data indicate RP-1 exhibits highest affinity for anionic bacterial lipids, with corresponding K_A_ (5.3×10^4^ M^−1^) and K_D_ (1.15×10^−7^ M) values, respectively. In this context, particularly at pH 7.5, RP-1 assumes a highly-ordered helical conformation. In contrast, in the eukaryotic zwitterionic lipid system, RP-1 assumes a largely random coil structure. Furthermore, RP-1 penetration into LUVs was significantly less in eukaryotic lipids as compared with prokaryotic systems. Observed reductions in peptide helicity and degree of penetration were corroborated by reduced associations of RP-1 with eukaryotic lipids as determined by FTIR and SPR. For example, the K_A_ was approximately 10-fold lower for eukaryotic versus prokaryotic lipid systems (Table 3). Similarly, RP-1 K_D_ was weak (pH 7.5) or undetectable (pH 5.5) in the presence of eukaryotic membrane mimetics. These data are highly consistent with our prior findings using NMR [10], and are consistent with efficacy results in the current study. Together, these observations support the hypothesis that RP-1 preferentially targets prokaryotic and mitochondrial membranes, where it adopts a well-ordered helix and penetrates into the bilayer in a pH dependent manner.

Prevailing models of amphipathic α-helical peptide mechanisms of action begin with the cationic antimicrobial peptide interacting with the anionic bacterial surface [2], [23]. Electrostatic affinity derives from intrinsic cationic charge from the peptide primary structure, and a composite of the electronegative phospholipid membrane composition and ΔΨ of a target organism. For example, bacteria typically have more electronegative lipid composition (e.g. POPG and cardiolipin) than mammalian-like cell membranes. Thus, ΔΨ of −120 mV to −150 mV are commonly seen in bacterial and mitochondrial membranes, whereas −60 mV to −80 mV are typical of eukaryotic cell membranes [2], [24]. In this model, RP-1 would initially have a relatively disordered conformation in aqueous environments prior to interaction with the anionic bacterial surface [2]. Only in context of prokaryotic lipid environments does such a peptide adopt a highly-ordered helical conformation, with an obtuse polar angle. These events may drive penetration and permeabilization of the membrane, dissipate ΔΨ, and ultimately result in massive de-energization or alternative mechanisms that lead to the eventual death of the bacterium [2], [23].

Current findings also shed new light on RP-1 interactions with and mechanisms of action against fungi. For example, RP-1 exerted anti-CA activity with a pH optimum of 5.5. These findings are consistent with the antifungal properties of α-helical peptides from other human kinocidins, such as human IL-8 [25]. Interestingly, RP-1 caused significant permeabilization, but only modest de-energization of CA, within the assay period. Moreover, all experimental measures of RP-1 interaction with eukaryotic lipids suggested minimal affinity or interaction at pH 5.5. These intriguing data suggest several possibilities for future investigation. For example, specific membrane constituents in CA (e.g. ergosterol) may uniquely influence RP-1 interactions to supersede predictions made using phosphatidylcholine and cholesterol (POPC∶CHO). This model is consistent with RP-1 permeabilization of the CA cell membrane, without significant membrane penetration [26]. Alternatively, RP-1 may rapidly permeabilize and penetrate the CA cell membrane, for eventual targeting of the fungal mitochondrion. Although the current studies did not detect significant ΔΨ dissipation by RP-1 in 1 h, our previous studies showed that RP-1 or other peptides significantly de-energize the CA mitochondrion at 2 h [8], [22]. Hence, the kinetic mechanisms of RP-1 may initially permeabilize the CA membrane as a means for the peptide to access and preferentially target the mitochondria within. Lastly, it is possible that the models used in the present studies limit further differentiation of eukaryotic rather than prokaryotic pathogen membranes. For example, while the zwitterionic membrane mimetic system employed herein reflects eukaryotic themes, it is more similar to mammalian rather than fungal lipid bilayers [2].

Finally, it is important to consider the potential indirect effects of pH on efficacy of RP-1 or other peptides. For example, it is likely that pH contributes to peptide susceptibility or resistance pathways of the target organisms themselves. For example, mild acidic conditions can induce stress response pathways in several bacterial and fungal pathogens [27], [28], [29]. In some cases, response to pH alters peptide susceptibility [8]. Alternatively, pH may have distinct effects on efficacy of different host defense peptides. For example, pH-influenced susceptibility to defensin-like peptides may be distinct from those of α-helical peptides such as RP-1. This concept would enable greater host defense coverage in dynamic physiologic and microbiologic contexts.

It is also important to consider the methodologic constraints of the current studies. In the prior NMR evaluation, RP-1 backbone trajectory and side chain conformations were evaluated at pH 5.0 in micelles. While it is possible to assess RP-1 structure by NMR at neutral or basic pH, amide H/D exchange is typically protein-specific. Thus, in more disordered proteins — where amide hydrogens are not H-bonded — exchange can occur more readily, whereas proteins with strong helical or sheet propensities have slower H/D exchange due to extensive N-H…O = C H-bonding. For this reason, RP-1 and like helical peptides are preferably analyzed at lower pH. The fact that RP-1 did not exhibit strong H^N^ signals in solution NMR at higher pH [10] affirms this concept. While beyond the scope of the present studies, such areas of investigation will likely contribute to a more complete understanding of host defense peptide structure-activity relationships.

In summary, the present results suggest that pH- and lipid-specific contexts mediate selective toxicity of antimicrobial peptide RP-1. These findings support the hypothesis that peptides such as RP-1 exert selective or optimal toxicity against microbial pathogens rather than host cells in relevant microenvironmental context. This interpretation is consistent with the concept of host defense peptide immunorelativity (AEGIS model), in which molecular defenses concatenate peptide structure-function relationships corresponding to anatomic or physiologic sites or conditions to optimize efficacy against cognate organisms therein [6].

## Materials and Methods

### Microorganisms

A panel of microorganisms comprising prototypic Gram-positive, Gram-negative, and fungal human pathogens was investigated. *Staphylococcus aureus* (SA) strains ISP479C (peptide-susceptible; [SA^S^]) and ISP479R (peptide-resistant [SA^R^]) exhibit distinct susceptibilities to human host defense peptides *in vitro* (e.g. platelet kinocidins [9]). Relative to parental strain ISP479C, the ISP479R strain has a mutation in the *snoD* gene which encodes the complex I NADH-ubiquinone oxidoreductase. Disruption of this gene leads to a reduced membrane potential and increased resistance to cationic antimicrobial peptides. *Salmonella typhimurium* (ST) strain 5996s (peptide-sensitive; [ST^s^]) and ATCC strain 14028 (peptide-resistant; [ST^R^]) is resistant to human defensin HNP-1 *in vitro*, and is hypervirulent in murine models of infection [14]. Likewise, *Candida albicans* (CA) strain 36082^S^ (peptide-susceptible; [CA^S^]) is a human clinical isolate, while strain 36082R (peptide-resistant; [CA^R^]) is derived from 36082S [30] and differs in expression of the *SSD1* corresponding to its peptide resistance phenotype [6]. Respective strains have otherwise equivalent genotypes and growth characteristics. All organisms were cultured to logarithmic phase in brain heart infusion broth (BHI) at 37°C (SA or ST) or 30°C (CA). Cells were harvested, washed, and briefly sonicated to assure singlet organisms. Organisms were quantified by spectrophotometry and adjusted to 1×10^6^ CFU in buffers as appropriate to specific experiments (see below).

### Antimicrobial Peptide RP-1

RP-1 is a synthetic antimicrobial peptide engineered based in-part on C-terminal microbicidal helices of platelet factor-4 (PF-4; CXCL4) family kinocidins from humans and other mammals [31], [32]. Our prior studies have demonstrated this domain manifests much of the direct microbicidal activity of kinocidins, recapitulating or exceeding the antimicrobial efficacy of respective holoproteins [25], [32]. RP-1 is comprised of 18 naturally occurring amino acid residues (MW, 2162.8) and has an isoelectric point (pI) of 10.8. Our previous NMR studies have substantiated that RP-1 has a highly-ordered α-helical conformation, particularly in context of bacterial lipid systems [10]. Mean hydrophobic moment (M_H_) and hydrophobicity of RP-1 were quantified using a consensus hydrophobicity scale (www.bbcm.univ.trieste.it/~tossi/HydroCalc), and its polar angle was determined using the Zidovetski method [33]. The potential affect of pH 5.5 versus 7.5 on net 3-dimensional conformation of RP-1 was assessed using the computational prediction of protonation software H++ (http://biophysics.cs.vt.edu/H/index.php).

### Comparative Antimicrobial Efficacy of RP-1

RP-1 efficacy against study organisms was evaluated using radial diffusion at pH 5.5 or 7.5 [1], [34]. In brief, logarithmic-phase organisms were inoculated (10^6^ CFU/ml) into buffered agarose (10 mM MES, pH 5.5 or 10 mM PIPES, pH 7.5), and poured into plates. Peptides (10 µg/well) were introduced into wells in the seeded matrix, and incubated for 3 h at 37°C. After overlay of nutrient medium, assays were incubated at 37°C or 30°C for bacteria or fungi, respectively. Zones of inhibition were measured at 24 h as the radius (mm) of complete or partial clearance subtracting the well radius. Independent experiments were repeated a minimum of two times.

### Comparative Mechanisms of RP-1 Action

Flow cytometry was used to measure two mechanisms of RP-1 action at pH 5.5 and 7.5: 1) MP; and 2) de-energization as measured by ΔΨ of bacterial cell membranes or fungal mitochondria. A FACSCalibur® instrument (Beckton Dickinson) was used to detect the following assay fluorophores: propidium iodide (PI; Ex_535 nm_/Em_620 nm_; Sigma, St. Louis, MO) to assess MP; and 3,3-dipentyloxacarbocyanine (DiOC_5_; Ex_484 nm_/Em_500 nm_; Invitrogen, Carlsbad, CA) to assess ΔΨ. For experiments, 2×10^7^ cells were incubated with peptide (10 µg/ml) in 1 ml of 10 mM PIPES (pH 7.5) or 10 mM MES (pH 5.5) for either 5, 30 or 60 min with shaking at 30°C (*C. albicans*) or 37°C (bacteria). At indicated time points, a stain buffer was added (DiOC_5_, 0.5 µM plus PI, 5.0 µg/ml, in 50 mM potassium-containing MEM (K^+^MEM; without phenol red, pH 7.2; Sigma). Cells were allowed to stain at room temperature for 15 min prior to analysis. Experiments included control cells exposed to well-established perturbants of MP (70% EtOH), ΔΨ (CCCP; 100 mM; Sigma), or K^+^MEM buffer alone. Flow cytometry was performed at 20°C in 10 mM K^+^MEM, with forward light scatter (FSC) data collected to ensure fluorescence was not an artifact of aggregation. The validity of these methods to assess MP or ΔΨ approaches the reliability of classical patch clamp methods [35], [36], [37]. For each study, the fluorescence from a minimum of 5,000 cells was acquired and plotted against FSC. Results from a minimum of two independent experiments done on different days were included in statistical analyses.

### RP-1 Binding to Membrane-Mimetic Liposomes

RP-1 interactions with membrane mimetic liposomes was determined by introducing peptide to solution-phase large LUVs in 10 mM PIPES (pH 5.5) or 10 mM HEPES (pH 7.5). Liposomes of palmitoyoleoyl-phosphatidylethanolamine and palmitoyloleoyl-phosphatidylglycerol (POPE / POPG, mole ratio of 3∶1) simulated the bacterial inner membrane. By comparison, liposomes of POPE-phosphatidylcholine and cholesterol (POPC / CHO, mole ratio 1.2∶1) simulated eukaryotic lipid membrane systems [11]. RP-1-liposome solutions were incubated by shaking for one hour at 25°C to reach equilibrium. The liposome-peptide solution was centrifuged at 100,000× g (30 min; Beckman Airfuge) to isolate liposomes with bound peptide. Concentration of RP-1 in lipid and aqueous phases was determined by transmission UV spectroscopy integrating a molar extinction coefficient of 1280 M^−1^ cm^−1^ for RP-1 at 280 nm [38]. Solution phase measurements were made directly in supernatant, whereas the lipid-peptide pellet was dispersed in 20 mM SDS-buffer to ensure optical clarity for UV spectroscopy. Peptide-to-lipid binding was expressed as an association constant K_a_, where [P] is molar concentration of RP-1 peptide in solution, [L] is the molar concentration of lipid, and [PL] is the molar concentration of peptide bound to lipid [39], as indicated in equation [1], below:
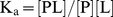
(1)


### Kinetic Assessment of RP-1-Lipid Interactions by Surface Plasmon Resonance

The kinetics of RP-1 interactions with lipid bilayers simulating bacterial versus eukaryotic membrane ensembles were evaluated by surface plasmon resonance (SPR) spectroscopy using a Biacore 3000 system (Biacore, Uppsala, Sweden). Liposomes (prepared as above) were deposited by drying lipid films from chloroform under high vacuum (12 h). Lipid films were then dispersed in PBS (pH 7.4) by vortexing and sonication to give a final lipid concentration of 20 mM. This suspension was extruded twenty times using a mini-extruder (1 µm polycarbonate filter; Avanti Polar Lipids, Alabaster, AL) to yield small unilamellar vesicles (SUV). Resulting SUV liposomes were deposited (rate, 2 µl/min) onto the surface of an L1 biosensor chip, forming lipid multilayers which were washed (10 mM NaOH at 10 µl/min) to ensure uniform lipid ensembles. For interaction kinetics, the running buffer was 50 mM Tris, 50 mM malate, and 150 mM NaCl adjusted to pH 5.5 [MES] or 7.5 [PIPES]. RP-1 interactions with lipid ensembles were determined by injecting samples at 50 µl/min for a 3 min association phase and a 3 min dissociation phase, each at 37°C. Binding affinities of RP-1 to the lipid multilayers were assessed by analysis of the SPR sensorgrams, with response units (RU) plotted as a function of time. Resulting sensorgrams were analyzed by BIAevaluation software (version 4.1) for curve fitting to determine mean on- and off-rate constants (K_on_ and K_off_) and dissociation constant (K_d_ = K_off_/K_on_) from measurements made at 1 µg RP-1/ml of buffer.

### RP-1 Conformation and Orientation in Membrane Mimetic Liposomes

The secondary structures and orientations of RP-1 were assessed in two distinct membrane mimetic liposomal systems that reflect the predominant lipids comprising bacterial versus eukaryotic membrane contexts [11]. Dispersions containing anionic lipid (POPE/POPG; mole ratio, 3∶1) emulated the bacterial membrane, while neutral lipid mixtures of POPC/cholesterol (mole ratio 1.2∶1) simulated eukaryotic membranes. The RP-1 peptide in ethanol was co-solvated with lipid systems in cholorform (ethanol∶choloroform, 1∶1, v∶v), followed by vacuum (10 mTorr) solvent removal under nitrogen. Resulting lipid-peptide films were dispersed in deuterated buffer (10 mM PIPES, pH 5.5 or 10 mM HEPES, pH 7.5) by vortex and repeated freeze-thaw cycles to obtain large unilamellar vesicles. Lipid-peptide samples were isolated by centrifugation (100,000× g; Beckman Airfuge TM). Pellets were re-suspended in 200 µl of deuterated water and transferred onto a germanium attenuated reflectance (ATR) crystal (50×20×2 mm; 45°; Pike Technologies, Madison, WI) to produce lipid-peptide films. Lipid-peptide matrices were then exposed to deuterium vapor to yield films ≥35% hydrated [40]. Infrared spectra were measured using a Bruker Vector FTIR spectrometer equipped with a deuterated triglycine sulfate detector (25°C). Resulting FTIR spectra were averaged for 256 scans at a gain of 4 and a resolution of 2 cm^−1^. Relative amounts of α-helix, β-turn, β-sheet, or random (disordered) structures were estimated using Fourier self-deconvolution (GRAMS/AI8, version 8.0, Thermo Electron Corp, Waltham, MA) and area of component peaks calculated using curve-fitting software (Igor Pro, version 1.6, Wavemetrics, Lake Oswego, OR) [41]. FTIR frequency limits were: α-helix (1662–1645 cm^−1^), β-sheet (1637–1613 and 1710–1682 cm^−1^), turn/bend (1682–1662 cm^−1^), and disordered or random (1650–1637 cm^−1^) [42].

To estimate the orientation of RP-1 in prokaryotic or eukaryotic membrane mimetics, gold wire polarizers (Perkin Elmer, Waltham, MA) were rotated from 0°–90° to obtain polarized IR spectra of each lipid-peptide film [43]. The insertion (or tilt) angle of RP-1 helical axes in the lipid multilayers was calculated based on the ratio RATR = A_∥_/A_⊥_ , with areas of absorbance at 0° and 90° polarizations for the amide I band centered at 1656 cm^−1^. These measures assumed a thick film approximation with the values for the electric field components of the evanescent wave to be E_x_ = 1.398, E_y_ = 1.516, E_z_ = 1.625, and an angle α = 39° for vibrational dipole relative to the molecular axis of the helix to derive an order parameter, S in equation ***[2]***, below [44]. The helical tilt angle, Θ was then derived from the order parameter using equation ***[3]***, below [44].
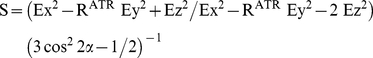
(2)

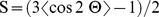
(3)


### Comparative Molecular Dynamics of RP-1 Interaction with Lipid Mimetic Systems

Computational studies of RP-1 interactions with lipid systems were conducted using GROMACS version 4.5.4 suite of molecular dynamics software [45]. Lowest energy atomic coordinates for RP-1 within SDS micelles (PDB accession code 2RLG; pH 5.0) were used as a starting structure within bacterial lipid environments, whereas the lowest energy conformer of RP-1 in DPC micelles (PDB accession code 2RLH; pH 5.0) was used as starting structure for simulated mammalian membranes [10]. Structural files for POPE, POPG, and POPC were accessed via the Tieleman website (http://moose.bio.ucalgary.ca). POPC parameters for the ffG53a6 force field were downloaded from (http://compbio.biosci.uq.edu.au/atb/) and POPE parameters were derived from conversion of POPC parent files to POPE values [46]. Topographies for POPG were those of Kukol [47]. Structure and parameter files for cholesterol were accessed through the ATB database (http://compbio.biosci.uq.edu.au/atb).

RP-1 peptide was initially docked onto the polar head group domain of pre-equilibriated POPE∶POPG (3∶1, mole∶mole) or POPC∶CHO (1∶1, mole∶mole) bilayers using Hyperchem (Gainsville, FL; version 7.52) with the CHARMM27 force field option [10], [48], [49]. The resulting ensemble was placed in a 65×65×100 Å periodic simulation box, solvated with SPC water and neutralized by appropriate counter ions. RP-1-lipid ensembles were optimized by lateral translation for molecular packing of lipids and peptide using established Perl script function, INFLATEGRO (http://moose.bio.ucalgary.ca/). Since there is no direct way to explicitly adjust the pH in the Gromacs force field environment, pdb2gmx (implementing the –lys option) was used to alter the degree of protonation of the lysine side chains to emulate differences in pH in the simulations [50]. Optimal lipid bilayer-peptide ensembles were then refined using Polak-Ribiere conjugate gradient minimization, and the systems were solvated by constraining the peptide over 20 psec of pre-run dynamics at 311 K. Pre-dynamics were followed by 50 nsec of molecular dynamics at 311 degrees K utilizing the ffG53a6 force field option (GROMACS) without constraints to achieve thermodynamically reasonable approximation of equilibrium structure for a given construct in each lipid bilayer system. Model structures for molecular graphics figures were rendered using PyMOL (version 0.99; http://www.pymol.org). The comparison of side chain conformations of simulated versus NMR peptide structures in various environments were analyzed using SCit (http://bioserv.rpbs.jussieu.fr/cgi-bin/SCit; [51]).

### Statistical Analysis

Differences in experimental outcomes were compared by student *t* or chi^2^ test as appropriate for specific data sets. Bonferroni correction was applied as appropriate for non-parametric data. ***P*** values≤0.05 (95%) were considered significant.

## Supporting Information

Figure S1 **Quantitative analysis of RP-1 on membrane energetics (DiOC5) and membrane permeabilization (PI) in ST, SA and CA.** Data are normalized to stained control cell fluorescence and expressed as percent of control (dashed line is equivalent to 100%). Time points: 5, 30 and 60 min. Data shown represent exposure to either ethanol (yellow), CCCP (red) or RP-1 (blue). Data represent mean ± one standard deviation for a minimum of two independent experiments. Statistical significance (***P***<0.05) indicated by asterisk. Data were generated using FCS Express software (version 3.0; De Novo; Los Angeles, CA).
(TIF)

Figure S2 **Secondary structure estimation as measured by FTIR spectroscopy.** Relative amounts of α-helix, β-turn, β-sheet, or random (disordered) structures were estimated by Fourier self-deconvolution (GRAMS/AI8, version 8.0, Thermo Electron Corp, Waltham, MA) and area calculations of component peaks calculated with curve fitting software (Igor Pro, version 1.6, Wavemetrics, Lake Oswego, OR). The FTIR frequency limits used for structures were: α-helix (1662–1645 cm^−1^), β-sheet (1637–1613 and 1710–1682 cm^−1^), turn/bend (1682–1662 cm^−1^), and disordered or random (1650–1637 cm^−1^).
(TIF)

Table S1 **Differences in experimental outcomes were compared by student t test.** P values≤0.05 (95%) were considered to be significant and are indicated in red.
(DOC)
