# Supplementary figures and images for: Context Mediates Antimicrobial Efficacy of Kinocidin Congener Peptide RP-1
Source: PLoS One. 2011 Nov 4;6(11):e26727. doi: 10.1371/journal.pone.0026727 (PMC3208557; doi:10.1371/journal.pone.0026727)

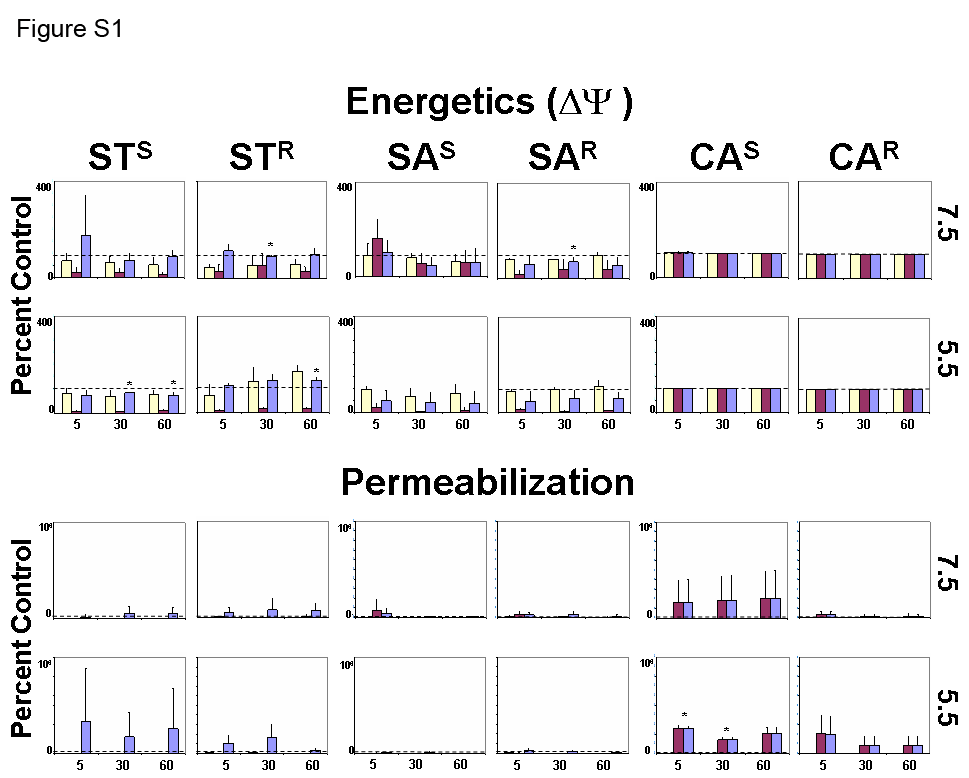

Supplement: Figure S1 — Quantitative analysis of RP-1 on membrane energetics (DiOC5) and membrane permeabilization (PI) in ST, SA and CA. Data are normalized to stained control cell fluorescence and expressed as percent of control (dashed line is equivalent to 100%). Time points: 5, 30 and 60 min. Data shown represent exposure to either ethanol (yellow), CCCP (red) or RP-1 (blue). Data represent mean ± one standard deviation for a minimum of two independent experiments. Statistical significance (P<0.05) indicated by asterisk. Data were generated using FCS Express software (version 3.0; De Novo; Los Angeles, CA). (TIF) [file pone.0026727.s001.tif]

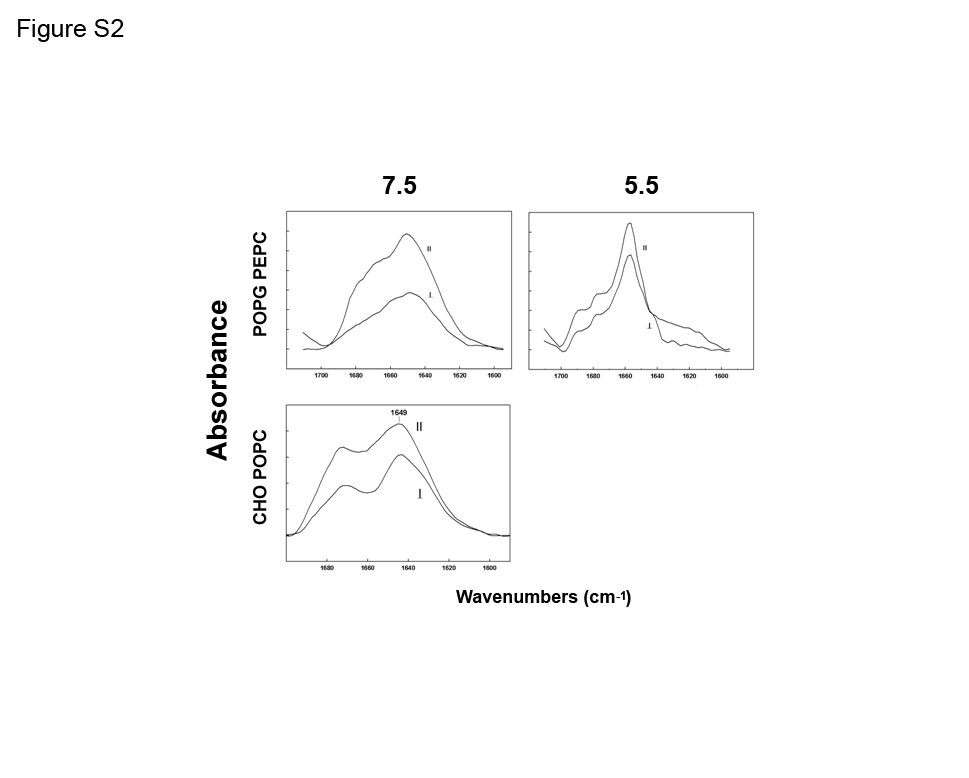

Supplement: Figure S2 — Secondary structure estimation as measured by FTIR spectroscopy. Relative amounts of α-helix, β-turn, β-sheet, or random (disordered) structures were estimated by Fourier self-deconvolution (GRAMS/AI8, version 8.0, Thermo Electron Corp, Waltham, MA) and area calculations of component peaks calculated with curve fitting software (Igor Pro, version 1.6, Wavemetrics, Lake Oswego, OR). The FTIR frequency limits used for structures were: α-helix (1662–1645 cm−1), β-sheet (1637–1613 and 1710–1682 cm−1), turn/bend (1682–1662 cm−1), and disordered or random (1650–1637 cm−1). (TIF) [file pone.0026727.s002.tif]
