# Supplementary material for: Context Mediates Antimicrobial Efficacy of Kinocidin Congener Peptide RP-1
Source: PLoS One. 2011 Nov 4;6(11):e26727. doi: 10.1371/journal.pone.0026727 (PMC3208557; doi:10.1371/journal.pone.0026727)
Supplement: Table S1 — Differences in experimental outcomes were compared by student t test. P values≤0.05 (95%) were considered to be significant and are indicated in red. (DOC) [file pone.0026727.s003.doc]

**Table S1.** Statistical analysis matrix of antimicrobial efficacy comparing microorganism type, strain and pH.

|  | **ST^S^5.5** | **ST^R^5.5** | **ST^S^7.5** | **ST^R^7.5** | **SA^S^5.5** | **SA^R^5.5** | **SA^S^7.5** | **SA^R^7.5** | **CA^S^5.5** | **CA^R^5.5** | **CA^S^7.5** | **CA^R^7.5** |
| --- | --- | --- | --- | --- | --- | --- | --- | --- | --- | --- | --- | --- |
| **ST^S^5.5** | - |  |  |  |  |  |  |  |  |  |  |  |
| **ST^R^5.5** | 0.01 | - |  |  |  |  |  |  |  |  |  |  |
| **ST^S^7.5** | 0.03 | 0.0001 | - |  |  |  |  |  |  |  |  |  |
| **ST^R^7.5** | 0.55 | 0.01 | 0.08 | - |  |  |  |  |  |  |  |  |
| **SA^S^5.5** | 0.003 | 0.08 | 0.0001 | 0.002 | - |  |  |  |  |  |  |  |
| **SA^R^5.5** | 0.003 | 0.005 | 0.0001 | 0.003 | 0.02 | - |  |  |  |  |  |  |
| **SA^S^7.5** | 0.4 | 0.005 | 0.004 | 0.18 | 0.0007 | 0.0005 | - |  |  |  |  |  |
| **SA^R^7.5** | 0.45 | 0.08 | 0.03 | 0.323 | 0.002 | 0.02 | 0.63 | - |  |  |  |  |
| **CA^S^5.5** | 0.0002 | 0.19 | 0.0001 | 0.0001 | 0.005 | 0.0001 | 0.0001 | 0.01 | - |  |  |  |
| **CA^R^5.5** | 0.002 | 0.859 | 0.0001 | 0.001 | 0.036 | 0.001 | 0.0008 | 0.02 | 0.22 | - |  |  |
| **CA^S^7.5** | 0.02 | 0.31 | 0.001 | 0.02 | 0.66 | 0.37 | 0.01 | 0.04 | 0.1 | 0.19 | - |  |
| **CA^R^7.5** | 0.03 | 0.38 | 0.0009 | 0.02 | 0.84 | 0.26 | 0.01 | 0.058 | 0.14 | 0.19 | 0.81 | - |

Differences in experimental outcomes were compared by student t test. P values ≤ 0.05 (95%) were considered to be significant and are indicated in red.
